# Bases moleculares de la patogénesis del ameloblastoma: Una revisión

**DOI:** 10.21142/2523-2754-1203-2024-212

**Published:** 2024-09-17

**Authors:** Grecia Lourdes Riofrio Chung, Tania Lisseth Santos Tucto, Angela Quispe-Salcedo

**Affiliations:** 1 Facultad de Odontología, Universidad Nacional Mayor de San Marcos. Lima, Perú. grecia.riofrio.06@gmail.com Universidad Nacional Mayor de San Marcos Facultad de Odontología Universidad Nacional Mayor de San Marcos Lima Peru grecia.riofrio.06@gmail.com; 2 Facultad de Medicina, Universidad Científica del Sur. Lima, Perú. tsantos@cientifica.edu.pe Universidad Científica del Sur Facultad de Medicina Universidad Científica del Sur Lima Peru tsantos@cientifica.edu.pe; 3 Knowledge Community “Sustainable Innovation in Dentistry”, Universidad Federico Villareal. Lima, Perú. Universidad Nacional Federico Villarreal Knowledge Community “Sustainable Innovation in Dentistry” Universidad Federico Villareal Lima Peru; 4 Division of Anatomy and Cell Biology of the Hard Tissue. Niigata University Graduate School of Medical and Dental Science. Niigata, Japón.aquispesa@dent.niigata-u.ac.jp Niigata University Division of Anatomy and Cell Biology of the Hard Tissue Niigata University Graduate School of Medical and Dental Science Niigata Japan aquispesa@dent.niigata-u.ac.jp

**Keywords:** ameloblastoma, reabsorción ósea, proteína SHH humana, sistema de señalización de la quinasa MAP, biología molecular, ameloblastoma, bone resorption, SHH protein human, MAP kinase signaling system, molecular biology

## Abstract

El ameloblastoma es un tumor odontogénico benigno muy frecuente y altamente invasivo. Su patogénesis aún no está totalmente esclarecida.

**Objetivo:**

Describir los hallazgos moleculares más importantes que fomentan la actividad proliferativa del ameloblastoma y los factores involucrados que promueven la invasión a los tejidos óseos circundantes.

**Metodología:**

Se realizó una búsqueda de evidencia científica a través de las bases de datos: ScienceDirect, Medline, Wiley, Web of Science and Google Scholar. Se revisó 32 artículos, cuyos criterios de inclusión fueron que hayan sido publicados en idioma inglés y español; estudios descriptivos y analíticos, revisiones narrativas y sistemáticas publicadas desde enero de 2015 hasta junio de 2021. Se excluyeron las cartas al editor.

**Resultados:**

Los hallazgos biológicos moleculares que permiten la invasión de ameloblastoma hacia tejidos circundantes mediante la alteración de vías RANK/RANKL/OPG, factor de crecimiento transformante beta (TGF-β), vía de las Wnt / β-catenina y las metaloproteinasas de matriz, además sus alteraciones en vías de MAPK y SHH las cuales permiten la proliferación y desarrollo tumoral del ameloblastoma.

**Conclusiones:**

Estos hallazgos son fundamentales para una mejor comprensión de las vías involucradas en la patogénesis del ameloblastoma.

## Abstract (en)

Ameloblastoma is the most common and highly invasive benign odontogenic tumor. Its pathogenesis is not yet fully understood.

**Objective:**

To describe the most important molecular findings that promote the proliferative activity of ameloblastoma and the factors involved that encourage invasion into surrounding bone tissues.

**Methodology:**

A search for scientific evidence was conducted through the following databases: Science Direct, Medline, Wiley, Web of Science, and Google Scholar. A total of 32 articles were reviewed, with inclusion criteria being articles published in English and Spanish; descriptive and analytical studies, narrative and systematic reviews published from January 2015 to June 2021. Letters to the editor were excluded.

**Results:**

The biological molecular findings that allow ameloblastoma to invade surrounding tissues involve alterations in the RANK/RANKL/OPG pathways, transforming growth factor beta (TGF-β), Wnt/β-catenin pathway, and matrix metalloproteinases, as well as alterations in MAPK and SHH pathways that facilitate the proliferation and tumor development of ameloblastoma.

**Conclusions:**

These findings are fundamental for a better understanding of the pathways involved in the pathogenesis of ameloblastoma.

## INTRODUCCIÓN

El ameloblastoma es un tumor odontogénico epitelial benigno, localmente invasivo al hueso adyacente ^1^, derivado de las células residuales de la lámina dental, restos celulares de Malassez o de la vaina de Hertwig, células del órgano del esmalte en desarrollo o quistes odontogénicos ^2^^,^^3^. 

La nueva clasificación de la Organización Mundial de la Salud (OMS), de 2017, subdivide a los ameloblastomas según sus variantes clínico-patológicas en ameloblastoma uniquístico, ameloblastoma multiquístico y tipo extraóseo o periférico ^4^. Esta patología representa, aproximadamente, el 1% del total de tumores orales y del 11 al 18% de los tumores odontogénicos ^5^^,^^6^. Tiene una incidencia de 0,5 casos nuevos por un millón de personas al año ^7^^,^^8^, con una mayor prevalencia en países en vía de desarrollo ^9^^-^^11^. Por lo general, los pacientes que presentan esta neoplasia tienen entre 30 a 50 años de edad, pero puede manifestarse en cualquier etapa de vida, sin diferencias de frecuencia entre los géneros ^12^. Aproximadamente, el 80% de casos se localizan en la mandíbula, siendo más frecuente la región de la tercera molar ^2^^,^^13^^,^^14^. Además, su patrón de crecimiento lento origina una expansión o perforación de las corticales óseas, y si no es tratado puede crecer de forma excesiva, hasta ocasionar deformidades faciales ^1^. 

La etiología del ameloblastoma aún es desconocida; aunque se ha sugerido el consumo del tabaco como un posible factor de riesgo ^4^. En los últimos años, la innovación en las técnicas de biología molecular ha permitido explicar la patogénesis del ameloblastoma sobre las bases de la clonación, el control del ciclo celular, la apoptosis, la supresión tumoral, los mecanismos osteoclásticos, la actividad de la matriz de metaloproteinasa y las vías de señalización ^3^. Sin embargo, aún queda mucho por conocer acerca del origen y desarrollo de esta patología. Por lo tanto, esta revisión se ha centrado en describir los hallazgos moleculares más importantes que fomentan la actividad proliferativa del ameloblastoma y los factores involucrados en la interacción entre células estromales y tumorales que promueven la invasión a los tejidos óseos circundantes.

## MATERIALES Y MÉTODOS

**Figura 1:**
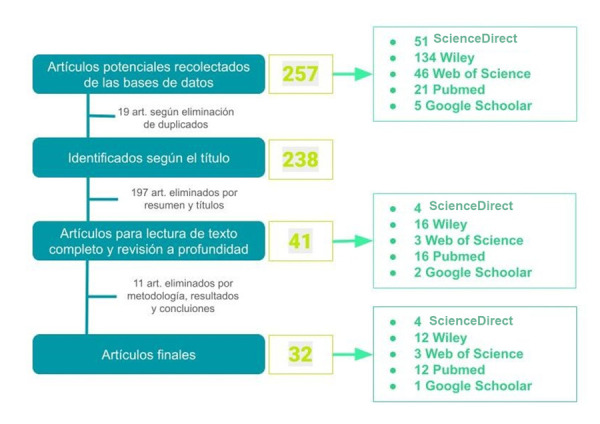
Visualización del proceso de búsqueda de literatura en bases de datos (ScienceDirect, Pubmed, Wiley, Web of Science y Google Scholar)

Se realizó una búsqueda de evidencia científica a través de las bases de datos: ScienceDirect, Medline, Wiley, Web of Science y Google Scholar utilizando los operadores boleanos OR, AND y NOT, y los descriptores “ameloblastoma”; [MeSH Terms], “Molecular biology”; [All Fields] y “dentistry”; [All Fields]. El total de artículos encontrados inicialmente fue de 257, de los cuales 51 fueron extraídos de ScienceDirect, 134 de Wiley, 46 de Web of Science, 21 de Pubmed y 5 en Google Scholar. En primer lugar, se eliminaron los duplicados y el total se redujo a 238; luego se analizó el título y se descartaron los artículos que no estaban relacionados con el tema de estudio, con lo cual quedaron 67. Posteriormente, se leyeron los resúmenes y se excluyeron los artículos que no examinaron las variables o no tenían resultados concluyentes, y se obtuvo 41 artículos. Finalmente, estos fueron analizados por completo y se eliminaron aquellos que no describían detalladamente la metodología de trabajo, los resultados o las conclusiones, lo que dejó un total de 32 artículos disponibles para esta revisión (Figura 1).

Los criterios de inclusión fueron los siguientes: artículos publicados en idioma inglés y español; estudios descriptivos y analíticos, revisiones narrativas y sistemáticas publicadas desde enero de 2015 hasta junio de 2021. Se excluyeron las cartas al editor.

## RESULTADOS

### Resorción ósea y osteoclastogénesis

Histológicamente, el ameloblastoma se compone de parénquima y estroma. El parénquima es el conjunto de células propias del tumor de origen epitelial, y el estroma, el soporte compuesto por tejido conjuntivo. En los últimos años, se ha demostrado que el estroma también ayuda a proporcionar un medio adecuado para la invasión y el crecimiento del tumor ^15^. Por lo tanto, es posible que los eventos moleculares que suceden en la interacción de células parenquimales y estromales del ameloblastoma favorezcan su invasión en los tejidos óseos adyacentes. La resorción ósea mediada por osteoclastos es un mecanismo fundamental para el avance del ameloblastoma. La diferenciación osteoclástica está regulada principalmente por el factor nuclear activador del receptor kappa β (RANK), el ligando del factor nuclear activador del receptor kappa β (RANKL) y la osteoprotegerina (OPG); por ende, referimos a este conjunto como el sistema RANK/RANKL/OPG. La unión de RANKL a su receptor de membrana RANK promueve la diferenciación de preosteoclastos a osteoclastos maduros, lo que da como resultado el proceso de reabsorción ósea; mientras que la OPG es un receptor señuelo, que inhabilita la interacción entre el RANKL y el RANK, lo cual inhibe la resorción y mantiene el equilibrio ^2^^,^^16^. 

El comportamiento clínico del ameloblastoma ha sido relacionado con una desregulación de la triada RANK/RANKL/OPG. Diversos estudios han demostrado que los ameloblastomas presentan una mayor expresión de RANK y RANKL en comparación con los queratoquistes odontogénicos, hecho que explica la mayor capacidad de invasión ósea del ameloblastoma ^17^^,^^18^. A nivel biológico, se ha señalado una alta expresión de RANKL en células del ameloblastoma o en el estroma ^6^^,^^18^, acompañado de una menor expresión de OGP, lo que ocasiona mayor liberación de forma soluble de RANKL, favorece una mayor diferenciación osteoclástica y, consecuentemente, el aumento del proceso de reabsorción ^16^^,^^17^^,^^19^.

### Citocinas inflamatorias

La expresión de RANKL puede ser desencadenada por factores de reabsorción ósea, tales como la presencia de interleucina 1 alpha (IL-1α), interleucina 6 (IL-6) e interleucina 8 (IL-8) o factor de necrosis tumoral- alpha (TNF-α) mediante la interacción de células tumorales y los fibroblastos estromales ^6^^,^^12^^,^^20^. La IL-8 es una quimiocina que se relaciona con el crecimiento tumoral, angiogénesis y metástasis. Según Liu *et al*. ^6^, se ha reportado que la IL-8 es secretada por las células del estroma de la médula ósea e inducida por el TNF-α, derivado de las células tumorales del ameloblastoma, en la que IL-8 puede activar la formación directa a los osteoclastos y estimular RANKL a nivel de los fibroblastos estromales del tumor. Por otro lado, el TNF-α producido por células inmunes es una citocina importante en la osteoclastogénesis, producida en condiciones inflamatorias. El TNF-α media la activación directa de osteoclastos y actúa como sinergista al regular al alza la expresión de RANKL o aumentar la producción de citocinas inflamatorias, como IL-6 e IL-8, en la que IL-6 se ha asociado con proliferación y motilidad de células propias del tumor ^21^. Igualmente, la IL-1α es una citocina que permite la expresión de otras moléculas inflamatorias y citocinas, expresada en el parénquima del ameloblastoma ^22^; además, puede inducir la producción de IL-6 e IL-8 por parte de las células del estroma ^20^. 

La tendencia de una mayor expresión de factores promotores de osteólisis se relaciona con el carácter invasivo y agresivo del ameloblastoma, ya que el tumor necesita un ambiente propicio para su proliferación.

### El rol de la superfamilia del factor de crecimiento transformante beta (TGF-β)

La superfamilia del factor de crecimiento transformante beta (TGF-β) abarca un gran número de moléculas relacionadas con funciones celulares como la proliferación, diferenciación, motilidad, angiogénesis y apoptosis; funciones que también están implicadas en los procesos de tumorogénesis ^23^. La TGF-β es una citocina que produce en el hueso efectos pleiotrópicos sobre los osteoblastos y osteoclastos para regular la remodelación ósea ^24^. Además, se sabe que los miembros de la superfamilia TGF-β funcionan como proteínas que participan en procesos de proliferación diferenciación y función de células óseas, entre ellos la osteoclastogénesis ^25^. Los genes afines con la vía de señalización TGF-β se han expresado de forma significativa en el ameloblastoma. Kondo *et al*. ^26^ señalan que los genes TGF- β1, TGF- β3 aumentaron significativamente en el ameloblastoma, mientras que Karathanasi *et al*. ^23^ hallaron una mayor expresión citoplasmática de TGF- β1, que puede estar asociada con un fenotipo menos diferenciado y una mayor proliferación, lo que estimula la progresión del tumor. 

Takebe *et al*. ^15^ encontraron que la expresión de TGF-β y BMP-4, otro miembro de la familia TGF-β, se asocia con células precursoras positivas para RANKL. Esto indica que TGF-β actúa como cofactor de RANKL ^12^ y promueve la osteoclastogénesis en el microambiente del ameloblastoma. También, TGF-β y el interferón-γ (IFN-γ) son factores que actúan de forma cooperativa para regular el aumento de los niveles de IL-6 y metaloproteinasa de matriz-9 (MMP-9), mediados por TNF-α, tomando en cuenta que este último factor aumenta los niveles de RANKL. Por ende, estos hallazgos sugieren que el TGF-β y el IFN-γ juegan un papel importante en la resorción ósea del microambiente ameloblastoma ^27^. 

Por otro lado, la interacción entre las células tumorales y células estromales de la médula ósea aumenta la secreción de activina A, otro miembro de la superfamilia TGF-β, el cual cumple un papel fundamental como cofactor del RANKL al aumentar la cantidad de osteoclastos. Sin embargo, se ha señalado que, en ausencia de activina A, la formación de osteoclastos no disminuyó de forma significativa, lo que supone que es un sinergista de RANKL ^6^.

### Alteraciones en la vía de señalización Wint beta catenina (Wnt/β-catenina) 

La vía de señalización Wint beta catenina (Wnt/β-catenina) está conformada por 19 genes y es importante en la odontogénesis. Dentro de esta vía existen dos grupos, siendo el más común la vía canónica, la cual es activada en su mayoría por la proteína Wint 1 (Wnt1) e inactivada por Wint 5a (Wnt5a) ^28^^,^^29^. Wnt 1 es una proteína fundamental en esta vía, considerada el activador más potente en la vía, pues al hacerlo induce la acumulación de β-catenina en el citoplasma, lo que permite su translocación nuclear, en la cual codificará genes para su expresión. Esta vía de señalización tiene funciones biológica como la de la proliferación celular, morfogénesis y regulación del ciclo celular^30^^,^^31^, por lo que anomalías en la vía de regulan estas funciones y promueven la aparición de tumores. Se ha informado que la activación anormal Wnt1 puede sobre expresar los genes diana en la vía Wnt/ β-catenina, lo que causa un crecimiento celular excesivo ^30^. 

En el ameloblastoma, Wang *et al*. ^30^ encontraron que la expresión fue altamente positiva para Wnt1 en el 84,5% de los casos. Los resultados de inmunohistoquímica mostraron que fue significativamente alto (p < 0,05) respecto de los tejidos normales orales, además de expresarse solo a nivel citoplasmático de las células tumorales. No obstante, también se expresó en los alrededores de los nidos de las células del ameloblastoma y en la capa de células reticulares estrelladas. 

Dutra *et al*. ^31^ detectaron Wnt1 en el 64,4% de los ameloblastomas, y la β-catenina mostró tinción citoplasmática en el 70,6% del epitelio del ameloblastoma. Huat *et al*. ^32^ hallaron una sobre expresión de ligando Wnt1 en la mayoría de las variantes histológicas del ameloblastoma. Todos estos resultados sugieren que Wnt1 es una molécula de señalización clave para el desarrollo del ameloblastoma. 

Por otro lado, la vía de señalización de Wnt/β-catenina participa en el desarrollo del ameloblastoma, mediante la regulación de la expresión de MMP y RANKL, lo que afecta la diferenciación de osteoclastos ^12^. Yang *et al*. ^33^ mencionan que la elevada presión hidrostática en los ameloblastomas incrementa la expresión de metaloproteinasa de matriz - 2 (MMP-2) y MMP-9, mediante la activación de la vía Wnt/β-catenina de las células del ameloblastoma. 

### Las metaloproteinasas de matriz (MMPs) 

Existen múltiples informes sobre la participación de las MMPs en el ameloblastoma. Sin embargo, su importancia reside en la degradación de componentes de la membrana basal y la matriz extracelular, al promover la invasión de células tumorales a los tejidos adyacentes. También poseen un rol importante como reguladoras de la proliferación y diferenciación celular, la respuesta inmune y la homeostasis tisular ^8^^,^^34^. 

Entre los estudios realizados sobre la fisiopatología del ameloblastoma, se ha reportado la manifestación del MMP-2 por los fibroblastos del estroma del tumor; pero también se ha observado que las células del ameloblastoma o parenquimales producen MMP-2, aunque en menor cantidad ^18^^,^^35^. Además, que la manifestación del MMP-2 está relacionada con un comportamiento más invasivo del ameloblastoma ^18^, ya que es una gelatinasa que degrada el colágeno tipo IV (principal componente de la membrana basal) ^2^^,^^36^. 

Asimismo, se ha descrito la participación de MMP-9 en la invasión local del ameloblastoma. Esta se expresa en los fibroblastos del estroma y la células tumorales ^2^, descompone el colágeno tipo IV y la gelatina, componentes principales de la membrana basal y la matriz extracelular ^8^^,^^26^^,^^37^. Además, esta proteína participa en la angiogénesis, remodelación del hueso y posterior metástasis del tumor. Kelppe *et al*. ^8^ detectaron la presencia de MMP-9 en las células inflamatorias, células gigantes multinucleadas entre la infiltración inflamatoria, lo que demuestra su capacidad de promover los procesos inflamatorios activando citocinas como IL-6, IL-8 e IL-1α, que participarán en el proceso tumoral y la activación de los osteoclastos. Este proceso de osteoclastogénesis permite crear un espacio propicio para la expansión del ameloblastoma ^12^.

### La participación vía de las proteínas cinasas activadas por mitógenos (MAPK)

En condiciones normales, la vía MAPK permite la amplificación de moléculas relacionadas con los procesos de proliferación, crecimiento y supervivencia celular. El esquema de esta vía consiste en la unión de uno o más factores de crecimiento con sus respectivos receptores, lo que desencadena la activación de la primera línea de intermediarios citosólicos, representada por las superfamilia RAS de GTPasas, siendo las principales HRAS, KRAS y NRAS ^38^. Con ayuda de proteínas intermedias, logra la conversión de guanosina difosfato (GDP) a guanosina trifosfato (GTP), y RAS-GTP es la forma activa ^39^. Posteriormente, se activará la familia RAF, cuyas isoformas principales son A-RAF; B-RAF y C-RAF ^40^^-^^42^, que continúan la vía mediante la activación MEK y posterior ERK ^41^^-^^44^, para finalmente pasar a la fase de regulación de la transcripción/traducción de genes efectores ^42^^,^^45^.

Se ha descrito que la desregulación de la vía MAPK estaría dada por mutaciones activadoras en BRAF en mayor frecuencia ^4^^,^^46^. BRAF es una serina treonina/quinasa que participa en la señalización intracelular de receptores de membrana hacia factores de transcripción nuclear, mediante las cascada RAS/RAF/MEK/ERK ^47^. El oncogén BRAF V600 se expresa a causa de una mutación puntual en BRAF, con una transversión de las bases nitrogenadas timina-adenina y, por consiguiente, un reemplazo de valina por ácido glutámico a nivel de traducción ^3^^,^^48^. La mutación da como resultado una ganancia de funciones de la proteína BRAF; al permanecer constantemente activada, aumenta la fosforilación de ERK, emite señales para el aumento de la proliferación celular, la supervivencia y, finalmente, la transformación neoplásica ^46^^,^^49^. 

Múltiples estudios han informado sobre la participación de BRAF V600 en la patogenia del ameloblastoma, cuya frecuencia varía entre el 40% y el 82% ^4^. Kurppa *et al*. ^50^ reportaron la presencia de este oncogén en el 63% (15/24) de los ameloblastomas analizados, lo que sugiere la participación de la vía BRAF/MERK/ ERK en el desarrollo del tumor. Duarte *et al*. ^4^ hallaron una elevada repetición de BRAF V600 en el 82% de los ameloblastomas. Otros estudios han reportado que los ameloblastomas con mutaciones BRAF V600 están relacionados con pacientes jóvenes ^51^, se localizan con mayor frecuencia a nivel mandibular ^3^^,^^52^^,^^53^ y algunos lo han relacionado con un patrón histológico no plexiforme ^48^^,^^54^. 

También se ha señalado que los ameloblastomas que expresan BRAF-V600E podrían estar asociados a cambios metabólicos, como la disminución los niveles de glicerol, por un aumento en la tasa de glicólisis, y se ha reportado que el ameloblastoma presenta una sobreexpresión de transportador de glucosa 1 (GLUT-1) en la membrana y el citoplasma ^4^. Se sabe que, en el epitelio oral normal, la expresión de GLUT-1 básicamente está limitada a la membrana celular; sin embargo, en las displasias o tumores malignos, GLUT-1 cambia su expresión a membrana/citoplasma, por lo que los tumores con GLUT-1 expresados en la membrana se asocian con menores demandas de glucólisis y proliferación. Por el contrario, los tumores con mayor expresión de GLUT-1 a nivel de citoplasma pueden estar relacionados con mayores demandas de energía, mayor tamaño del tumor y un peor pronóstico ^55^^,^^56^; esto podría explicar la diferencia del comportamiento de las variantes histológicas del ameloblastoma. La presencia citoplasmática de GLUT-1 se ha reportado en mayor frecuencia en la variante sólida del ameloblastoma al compararla con la variante uniquística, ya que esta última se considera menos agresiva.

Asimismo, la patogenia del ameloblastoma se ha relacionado con una hiperactividad de ligandos asociados a la vía MAPK. Nakao *et al*. ^57^ sugieren que el factor de crecimiento de los fibroblastos (FGF), como el FGF-7 o el FGF-10, jugaría un rol importante en la proliferación de células del ameloblastoma mediante la vía MAPK. Cabe resaltar que la mayor expresión de los ligandos FGF-7 y FGF-10 tuvo una mayor expresión en células estromales, lo que podría indicar que estos actúan como agentes paracrinos en la proliferación del ameloblastoma. Además, se ha reportado que una mutación con ganancia de funciones del receptor del factor de crecimiento de fibroblastos 2 (FGFR2) activaría la vía MAPK de manera continua y desregulada ^51^^,^^54^, ya que el FGFR2 es uno de los receptores que activa el RAS. Por otro lado, la patogénesis del ameloblastoma también se ha asociado con una hiperactividad del receptor del factor de crecimiento (EGFR) ^50^, lo que ocasiona un aumento en los niveles de ERK fosforilada y promueve la proliferación del tumor.

### Alteraciones en la vía de señalización de Sonic Hedgehog (SHH)

La vía de señalización de Sonic Hedgehog (SHH) participa en la odontogénesis a través del gen SHH, que codifica la formación de la proteína del mismo nombre. Esta proteína también juega un rol muy importante en el desarrollo embrionario general en vertebrados e invertebrados, por lo que solo se encuentra activa en la etapa embrionaria y no en la vida adulta ^58^^,^^59^. Shh activa el complejo del receptor de membrana Patched 1 (PTCH 1) y Smoothened (SMO) ^59^. PTCH1 es una proteína de transmembrana de 12 dominios y SMO que se caracteriza por tener 7 dominios de transmembrana asociados a proteínas G ^38^^,^^39^. La unión de SHH al receptor PTCH 1 causa la inhibición de SMO y activa las proteínas Gli (glioma), las cuales codifican la expresión de genes diana, lo que da como resultado una mayor proliferación celular ^60^.

En los vertebrados existen tres proteínas Gli: Gli 1, Gli 2 y Gli3. Gli 1 tiene una función estimuladora, Gli 3 es un inhibidor y Gli2 posee ambas funciones. La vía del Shh estimula la expresión de genes tales como Gli 1, PTCH1, CCND1 (ciclina D1), regulador del ciclo celular, cMyc (proliferación celular y tumorogénesis) y proteína 2 de linfoma de células B (Bcl-2), por lo que esta vía de señalización está involucrada en actividades de regulación del ciclo celular ^59^. De forma regular, esta vía se encuentra frecuentemente inactiva, pero se ha descrito que las mutaciones de genes que estimulan esta vía o la sobreexpresión del ligando SHH están relacionadas con la formación de tumores, tales como como la ciclina D2 (CCND2) y ciclina E1 (CCNE1) ^61^, el regulador de la apoptosis Bcl-262 o NMyc que participa en la multiplicación celular ^63^, debido a que se altera el control del ciclo celular y se estimula la proliferación celular ^58^. 

En el caso del ameloblastoma, Kumamoto *et al*. ^64^ compararon la expresión de SHH en ameloblastomas con células del gérmenes dentarios y encontraron una sobreexpresión de SHH solo en las células del tumor. Esta comparación se realizó debido a que los gérmenes dentarios tienen un patrón histológico similar al ameloblastoma y, como la vía de señalización del SHH está relacionada con el desarrollo embrionario, es importante mencionar sus diferencias. Por otro lado, Araújo *et al*. ^65^ hallaron una sobreexpresión de genes como SMO, PTCH 1, Gli 1 y CCND1 asociados con la vía SHH en todos los tumores estudiados de ameloblastoma, lo que sugiere la participación de esta vía en el desarrollo de los diferentes tipos de ameloblastoma. Esto fue confirmado por Gültekin *et al*. ^66^ que, según las características histológicas y clínicas, describieron una mayor frecuencia en la expresión de SMO en ameloblastomas sólidos, localizados en maxila. Sweeney *et al*. ^54^ encontraron que casi el 50% de los ameloblastomas presentaban mutaciones activadoras de SMO, predominantemente en el patrón plexiforme y ubicados mayormente en la maxila.

## DISCUSIÓN

El ameloblastoma es un tumor odontogénico de crecimiento lento, pero localmente infiltrativo cuya etiología y fisiopatología no han sido completamente investigadas. Existe una similitud entre el ameloblastoma y el desarrollo del germen dentario, lo que podría deberse a que ambos derivan de células que participan en la embriogénesis del diente, como el epitelio de revestimiento del folículo dental, restos epiteliales de la lámina dental o células basales de la mucosa ^30^. Esto puede ser sustentado, ya que diversos autores han encontrado moléculas que participan en las vías de la odontogénesis, como Wnt/ β-catenina o la vía Sonic Hedhogh. Dichos hallazgos pueden ser claves para el desarrollo de nuevas bases diagnósticas, el pronóstico y una potencial molécula para el desarrollo de terapias dirigidas del ameloblastoma. El tratamiento estándar actual es quirúrgico, como la resección mandibular y de tejidos circundantes para evitar el riesgo de recurrencias, lo que origina problemas a la mayoría de los pacientes ^2^. Sin embargo, una vez identificada una molécula clave en el desarrollo o proliferación de esta patología, existe de por sí una posibilidad de tratamiento, ya que el principal beneficio de las terapias moleculares dirigidas es la reducción de morbilidad quirúrgica. Por lo tanto, es importante seguir investigando los factores causales y sus mecanismos patogénicos. 

Finalmente, el sintetizar los hallazgos ayuda a tener un panorama completo sobre los nuevos conocimientos que han ido apareciendo durante los últimos años y así usarlos para su respectiva aplicación clínica. Esta revisión resume las últimas actualizaciones de las bases moleculares del ameloblastoma y, a pesar de la limitación de la literatura, se ha podido avanzar en la comprensión de su fitopatología, al describir la implicancia de una diversidad de moléculas y alteraciones genéticas que influyen en la interacción del estroma y el parénquima durante el desarrollo y progreso del ameloblastoma. Es necesario incentivar el conocimiento de los mecanismos biológicos detrás de los hallazgos clínicos, especialmente entre el odontólogo general y los estudiantes de odontología, y promover al mismo tiempo la investigación formativa en patología oral desde el punto de vista biológico.

Limitaciones: Se incluyó únicamente artículos en idioma inglés y español, lo cual pudo excluir información relevante en otros idiomas.

## CONCLUSIONES

El ameloblastoma, aunque clasificado como un tumor odontogénico benigno, presenta características altamente invasivas que complican su manejo clínico. Este estudio resalta la importancia de las técnicas de biología molecular en la comprensión de su patogénesis, y revela alteraciones significativas en diversas vías moleculares, como RANK/RANKL/OPG, TGF-β, Wnt/β-catenina y metaloproteinasas de matriz. Estas alteraciones no solo facilitan la invasión del tumor en los tejidos circundantes, sino que contribuyen a su proliferación y desarrollo. Los hallazgos subrayan la necesidad de continuar investigando estos mecanismos moleculares para desarrollar estrategias terapéuticas más efectivas que puedan mejorar el pronóstico y tratamiento del ameloblastoma.

## Fuente de financiamiento:

Esta investigación no ha recibido fuente de financiamiento de ninguna agencia pública o comercial
